# miR-150 is downregulated in osteosarcoma and suppresses cell proliferation, migration and invasion by targeting ROCK1

**DOI:** 10.3892/ol.2022.13462

**Published:** 2022-08-17

**Authors:** Chang-Hui Li, Teng-Bo Yu, Hong-Wei Qiu, Xia Zhao, Chuan-Li Zhou, Chao Qi

Oncol Lett 13: 2191–2197, 2017; DOI: 10.3892/ol.2017.5709

Following the publication of the above paper, a concerned reader drew to the Editors' attention that the Transwell cell migration and invasion assay data shown in Fig. 3C appeared to overlap in a pair of the data panels within this figure; in addition, data that were strikingly similar to those included in this figure had already appeared in previously published papers written by different authors at different institutes. Moreover, there was an unexpected level of similarity regarding the control β-actin bands shown in the three western blot experiments in Fig. 4B, even though these experiments related to three different cell lines. Having considered the issues identified, the Editor of *Oncology Letters* has decided that this article should be retracted from the Journal on account of the previously published data, and uncertainties regarding other data in Figs. 3 and 4. The authors were asked for an explanation to account for these concerns, but the Editorial Office did not receive a reply. The Editor apologizes to the readership for any inconvenience caused.

